# Primary Budd-Chiari Syndrome With Right Atrial Extension: A Rare Presentation of Intrahepatic Cholangiocarcinoma

**DOI:** 10.7759/cureus.18935

**Published:** 2021-10-20

**Authors:** Ana Gonçalves

**Affiliations:** 1 Internal Medicine, Hospital Fernando Fonseca, Amadora, PRT

**Keywords:** liver malignancy, cholangiocarcinoma, intrahepatic cholangiocarcinoma, right atrium thrombus, budd-chiari syndrome

## Abstract

Budd-Chiari syndrome (BCS) is defined as hepatic venous outflow obstruction and can be classified as primary when the obstruction is due to a predominantly venous process, caused by multiple risk factors that lead to a prothrombotic state. We report a case of a primary BCS, with an exuberant thrombus extending from the supra-hepatic vein, via the inferior vena cava, to the right atrium, a rare form of presentation of intrahepatic cholangiocarcinoma (ICC).

## Introduction

Budd-Chiari syndrome (BCS) is a rare but fatal disease caused by an obstruction in the hepatic venous outflow tract, and it can be classified as primary, when the obstruction is due to a predominantly venous process, or secondary, when the compression or invasion of the veins is caused by an extrinsic process [1]. Most patients with BCS have an underlying condition that should be promptly investigated and, if possible, treated. Multiple risk factors have been identified and are often combined in the same patient [2]. The presentation and clinical manifestations are extremely varied, so clinicians must have a high level of suspicion, and consider BCS in any patient with acute or chronic liver disease [3].

## Case presentation

**Figure 1:**
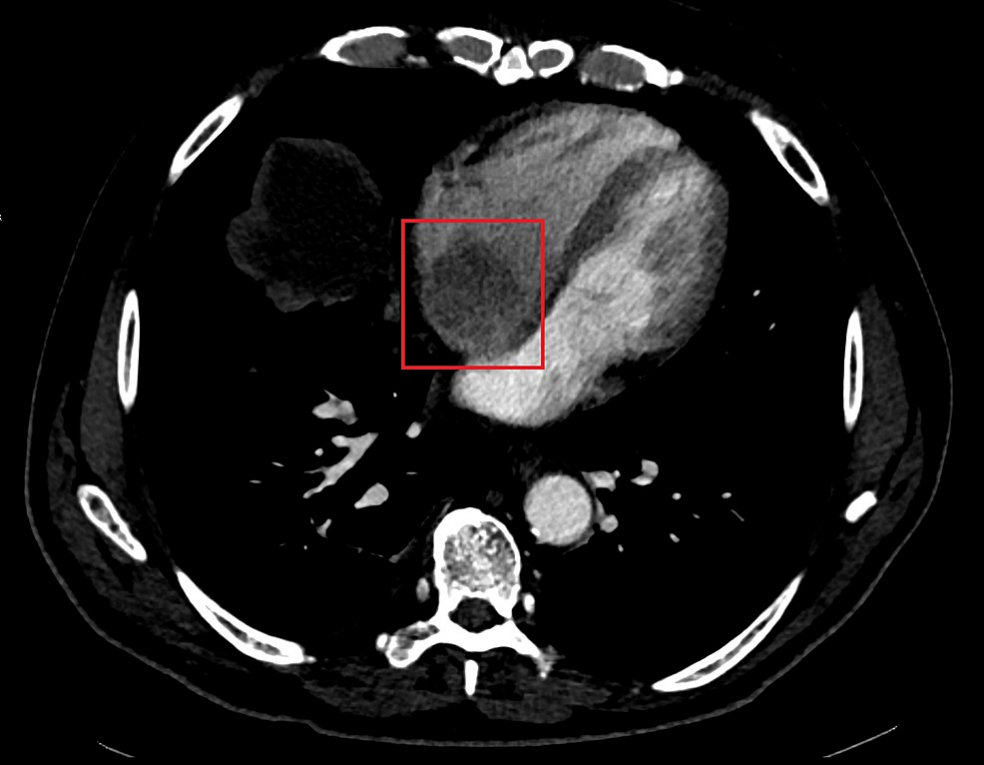
Abdominal CT scan showing the right atrium with thrombus (red box).

**Figure 2:**
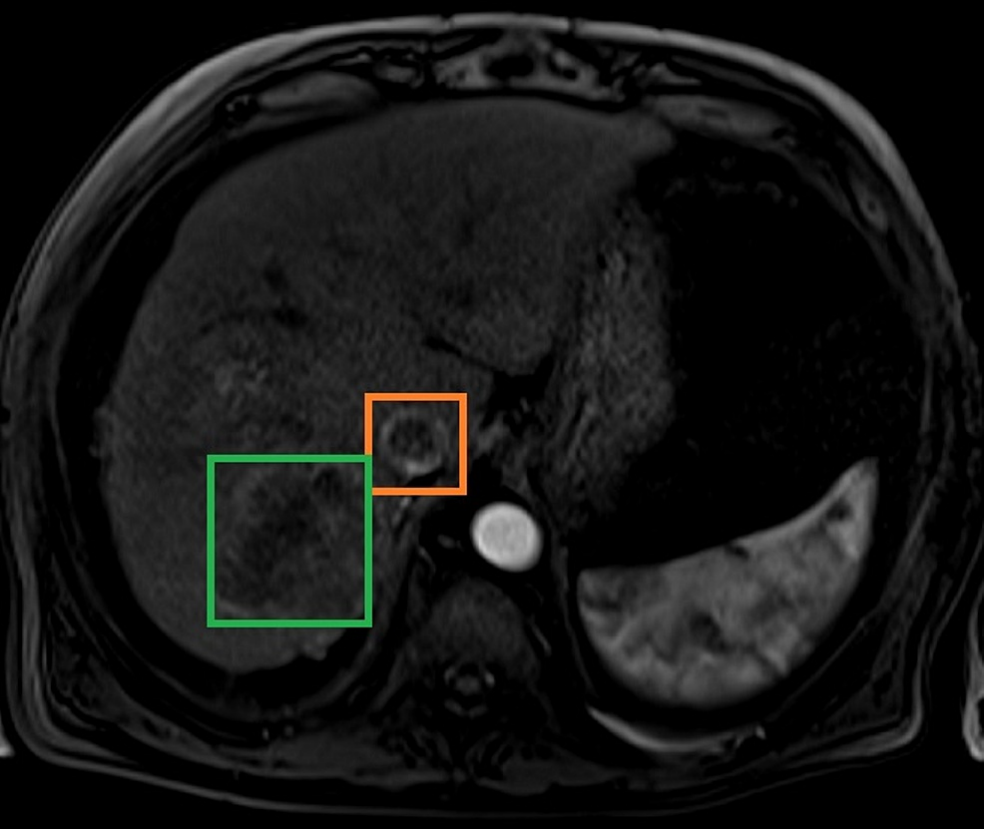
Abdominal MRI (T2) showing inferior vena cava with thrombus and hepatic tumor. Orange box: Inferior vena cava with thrombus; Green box: Hepatic tumor.

**Figure 3:**
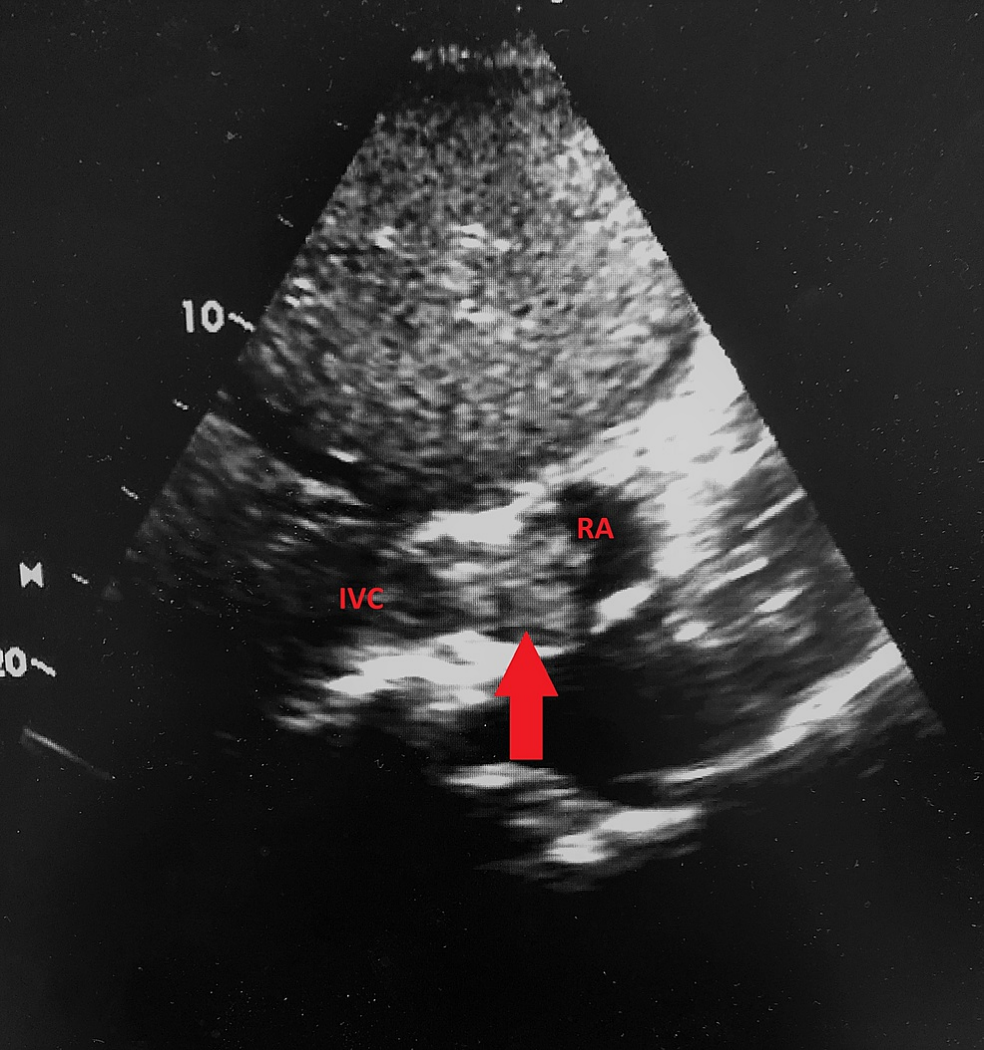
ECG (subcostal view) with right atrium thrombus (5 x 4 x 3 cm). Arrow: Thrombus; IVC: Inferior vena cava; RA: Right atrium.

We present a case of a 71-year-old male patient, admitted due to abdominal distension, pain in the right hypochondrium, and fatigue, developing over two months. He denied nausea, vomiting, anorexia, weight loss, jaundice, or other symptoms. On admission to the ED, he was conscious and oriented, with blood pressure: 120/73 mmHg, heart rate: 65 beats per minute, afebrile and eupneic with oxygen saturation: 98% in room air. Cardiopulmonary auscultation revealed rhythmic sounds, with a systolic right-sided heart murmur, and crackling rattles in both lungs. The abdomen was distended, painful on palpation, and with ascites. Blood tests revealed: aspartate aminotransferase (AST): 232 U/L, alanine aminotransferase (ALT): 121 U/L, total bilirubin: 1.06mg/dL, alkaline phosphatase 131 U/L and gamma-glutamyl transpeptidase (GGT): 182 U/L. Alpha-fetoprotein was 20 ng/mL. An abdominal Doppler ultrasound revealed moderate ascites and thrombosis of the portal vein and suprahepatic veins, along with a nodular hepatic lesion. Abdominal CT scan (Figure 1) and MRI (Figure 2) confirmed thrombosis of the portal vein, suprahepatic vein, and inferior vena cava extending to the right atrium, associated with a hepatic tumor. An ECG was performed to better characterize the intra-auricular thrombus (Figure 3). The diagnosis of intrahepatic cholangiocarcinoma (ICC) was confirmed by liver biopsy. Medical treatment was started immediately with anticoagulation and chemotherapy (gemcitabine and oxaliplatin), unfortunately, the patient had a poor therapy response, dying a few weeks later.

## Discussion

The clinical presentation of BCS may vary from a completely asymptomatic condition to fulminant liver failure. It depends on the extent of hepatic vein occlusion and whether venous collateral circulation has developed [1,4]. Patients with fulminant courses develop acute liver failure, jaundice, and hepatic encephalopathy. Subacute form of BCS is the most common, and usually progress in months with abdominal pain, hepatomegaly, and ascites. The chronic form is manifested with complications of cirrhosis [4].

ICC is a primary tumor, originating from the bile duct lining epithelium, frequently with an indolent course. Patients often have a history of dull right upper quadrant pain and weight loss. Some patients are asymptomatic, with the lesions being detected incidentally as part of the workup of abnormal liver blood tests [5]. This case configured a subacute form of BCS, as the clinical symptoms developed progressively over two months. As for classification, we are facing a primary BCS, since the obstructive event of the hepatic veins was a venous thrombotic process, triggered by ICC.

There are several cases in the literature of ICC with secondary BCS, related to tumor invasion of the hepatic veins, however primary BCS is rarely described [6].

BCS requires prompt diagnosis and treatment. As the presentation is highly variable, clinicians should consider it if the patient presents with acute liver failure or chronic liver disease [7]. Diagnosis can be made non-invasively using Doppler USG (sensitivity and specificity of 85%) [4], which is the technique of choice for initial investigation, however, contrast-enhanced CT and MRI can better demonstrate the necrotic areas of the liver [1,7].

In this case, workup with abdominal Doppler USG showed a nodular hepatic lesion along with thrombosis of the portal vein and suprahepatic veins. ICC usually presents as a malignant-appearing mass lesion in a non-cirrhotic liver, and the main differential diagnosis should always include a primary hepatocellular carcinoma (HCC) or metastatic adenocarcinoma [8]. Also, in ICC, the liver blood tests usually show elevated levels of alkaline phosphatase and GGT, whereas serum bilirubin levels and alpha-fetoprotein are normal [9]. Our patient presented with moderate elevation of transaminases, alkaline phosphatase, and GGT, which can be explained by both BCS and ICC. Normal levels of alpha-fetoprotein supported the diagnosis of ICC over HCC. The diagnosis was confirmed by liver biopsy.

Regarding BCS, it was possible with MRI to confirm the diagnosis and detect the extension of the thrombus from the vena cava to the right atrium, a rare form of presentation. 

Treatment of BCS is based on a stepwise management strategy that includes anticoagulation, correcting underlying disorders that predispose the development of a prothrombotic state, and complications of portal hypertension [10]. Patients without progressive liver necrosis (few symptoms, relatively normal liver function tests) and with ascites, require medical therapy alone. Patients with coagulopathy, encephalopathy, or hepatorenal syndrome (signs of poor prognosis), require immediate relief of the hepatic venous outflow tract obstruction, through thrombolytic therapy or angioplasty. In extreme cases, where prior therapy has failed, liver transplantation should be considered [3,4,7].

The prognosis of patients with BCS has improved in the past decades, due to a combination of faster diagnosis, new treatment modalities, and the routine use of anticoagulation [4,11].

## Conclusions

BCS has an extremely varied clinical course; therefore, clinicians must have a high level of suspicion and consider this diagnosis in any patient with acute or chronic liver disease. This case describes a rare form of presentation of an ICC through a primary BCS, with an exuberant thrombus from the suprahepatic veins to the right atrium, reminding us of the importance of always considering the presence of an underlying disorder in BCS, and consequently perform a full investigation to identify and treat the cause.
